# “It didn’t hurt me”: patients’ and providers’ perspectives on unsupervised take-home doses, drug diversion, and overdose risks in the provision of medication for opioid use disorder during COVID-19 in San Juan, Puerto Rico

**DOI:** 10.1186/s12954-024-01006-w

**Published:** 2024-04-25

**Authors:** Roberto Abadie, Celia B. Fisher

**Affiliations:** 1 School of Global and Integrative Studies, University of Nebraska-Lincoln, 839 Oldfather Hall, Lincoln, NE 68588 USA; 2 Center for Ethics Education, Fordham University, Rose Hill Campus, Dealy Hall, Room 117, New York City, NY 10023 USA

## Abstract

**Background:**

During the COVID−19 pandemic, clinics offering medication for opioid use disorder (MOUD) needed to rapidly introduce unsupervised take−home dosing, while relapsing patients and patients unable to enter treatment faced increased risks of fentanyl−related overdose deaths and other drug−related harms. Based on a qualitative study of people who inject drugs (PWID) receiving MOUD treatment and MOUD staff in Puerto Rico, this paper documents the lived experiences of patients and providers during this period and the risk perceptions and management strategies to address substance misuse and drug diversion attributable to unsupervised take−home−dose delivery.

**Methods:**

In−depth qualitative interviews were conducted with patients (N = 25) and staff (N = 25) in two clinics providing MOUD in San Juan, Puerto Rico, during 2022. Patients and staff were receiving or providing treatment during the pandemic, and patients reported injection drug use during the past thirty days.

**Results:**

Patients were overwhelmingly male (84%), unmarried (72%), and unemployed (52%), with almost half (44%) injecting one to three times a day. Mean time in treatment was 7 years. Staff had a mean age of 46 years with more than half of the sample (63%) female. The majority of patients believed that unsupervised take−home dosing had no significant effect on their treatment adherence or engagement. In contrast, providers expressed concerns over the potential for drug diversion and possible increased risks of patient attrition, overdose episodes, and poor treatment outcomes.

**Conclusion:**

This study underscores the importance of insider perspectives on harm−reduction changes in policy implemented during a health crisis. Of note is the finding that staff disagreed among themselves regarding the potential harms of diversion and changes in drug testing protocols. These different perspectives are important to address so that future pandemic policies are successfully designed and implemented. Our study also illuminates disagreement in risk assessments between patients and providers. This suggests that preparation for emergency treatment plans requires enhanced communication with patients to match treatments to the context of lived experience.

## Introduction

COVID-19 saw an increase in mental health issues and substance abuse among the general population [1, 2]. The effects of COVID-19 were particularly severe among people with OUDs. Limitations in health services during COVID-19 represented a serious challenge to people with OUDs because members of this population can be prone to conditions such as diabetes, cardiovascular problems, respiratory issues, or compromised immune systems. These vulnerabilities expose them to disproportionately high mortality risks [3]. Compounding these health risks, COVID-19 lockdown measures such as stay-at-home orders for nonessential workers and social distancing measures severely disrupted access to medication for OUD (MOUD) [4]. MOUD has shown positive effects, including reducing participation in illegal activities, drug use frequency, HIV risk behaviors, HCV transmission, and overdose episodes [5–9].

Thus, COVID−19 related stressors such as lack of income, isolation, and anxiety were compounded by changes in the drug supply, difficulties accessing harm−reduction resources, and stigma surrounding MOUD operations [10, 11]. Patients receiving MOUD treatment, particularly in the lockdown phase, during which most restrictions were implemented, faced increased risks of relapse, treatment discontinuation, overdose episodes, and deaths and other drug−related harms [12].

While the impact of COVID-19 on people with OUDs has been significant, people who inject drugs (PWID) and, in particular, Puerto Rican PWID bore a disproportionate percentage of overdose deaths [13–15]. Although epidemiological data is not yet available, it is likely that increased drug use frequency led not only to an increase in overdose episodes and deaths but also higher HIV and HCV (Hepatitis C virus) risks among this vulnerable population. PWID on the island of Puerto Rico faced the effects of COVID-19 superimposed on an already existing epidemic of HCV [16]. According to the last US Census, almost half of the Puerto Rican population (44%) live in poverty, three times the rate of the continental US [17]. Among PWID, poverty levels are even higher, with almost half having been homeless at some point during the past year [18]; homelessness disproportionately exposes them to COVID-19 risks.

Faced with the unprecedented pandemic situation, MOUD clinics in Puerto Rico, like their counterparts in the continental US, were forced to improvise and adapt in order to ensure continuous treatment services to a vulnerable and highly at−risk population [19–22]. Unsupervised take−home dosing for patients, along with a significant reduction or even a temporary elimination of drug testing, was introduced, and new enrollments were completely eliminated or reduced to a trickle. As a result of these changes and disruptions, patients who experienced relapses or those unable to enter treatment faced increased risks of fentanyl−related overdose deaths and other drug−related harms. Studies conducted on the US main land have provided mixed results on the success of take−home dose procedures during COVID−19. For example, staff at a California site were able to introduce take−home doses cautiously, making individualized risk/benefit assessments based on the probability that a patient would engage in misuse leading to overdose risks with current patients reporting increased autonomy and flexibility, leading to increased program engagement and patients that were not eligible demanding more transparency in the way decisions were made [23]. Another qualitative study, conducted in New Jersey, showed similar results, suggesting that some patients viewed positively the convenience, reduced travel expenses to the clinic, along with reduced stigma, but some resented the lack of other forms of support, such as food, clothing, or access to harm−reduction materials [24]. In other main land sites, there was evidence that for some patients unsupervised take−home dosing was never implemented or was implemented inconsistently [25] and that unhoused patients perceived take−home measures as impractical, unjust, and enhancing existing inequalities in treatment access [26]. In many MOUD sites in Puerto Rico, immediate restrictions on services posed by public health officials did not provide staff the opportunity to proceed cautiously or tailoring take−home doses to individual patient needs. In addition, harm reduction services on the Island were still suffering from the economic and health upheavals caused by Hurricane Maria when the pandemic hit. Thus, it is important to understand how patients and staff in Puerto Rico experienced unsupervised home delivery, disruptions at the clinics and the multiple services they were able to provide.

This paper aims to document the lived experiences of Puerto Rican MOUD patients and providers during the COVID−19 pandemic. Specifically, their risk perceptions and management strategies to address treatment needs, substance misuse and drug diversion attributable to unsupervised take−home−dose delivery during the pandemic.

The study was conducted among active PWID enrolled in MOUD treatment and staff at two clinics located in San Juan, Puerto Rico’s capital. San Juan and its metropolitan area concentrate almost two-thirds of the island’s 3.5 million people and contain the largest population of PWID and the majority of centralized MOUD resources. The capital was disproportionately affected by COVID-19, with close to 192,802 cases at the time of this submission [27].

PWID were and still are particularly vulnerable to overdose risks and other drug−related harms, and their views—as well as the perceptions of their providers—should be taken into account when designing harm−reduction strategies for a future pandemic.

## Methods

### Participants

PWID receiving MOUD and health care workers providing treatment were drawn from the same clinics in San Juan, Puerto Rico. One clinic was a relatively small, community-oriented, office-based buprenorphine site (Clinic A), and the other was a large Opioid Treatment Program (OTP) (Clinic B). Methadone provided at OTP clinics is classified by the federal government as a Schedule II controlled substance with high potential for abuse, while Buprenorphine is classified as a Schedule III controlled substance with lower potential for abuse compared with Schedule I and II, but still with potential for misuse. For this reason, the medications therapeutic use is strictly controlled by federal regulations, particularly in relation to take-home dosing requirements [28]. Before the pandemic, take-home doses were allocated based on a number of factors, such as the patients’ adherence to treatment, determined by regular urine tests and frequency of visits, as well as the need to comply with program rules and regulations. These regulations sought to balance the need to provide access to medication while minimizing the risk of drug diversion and misuse. During COVID-19, take-home dosing requirements were relaxed, moving all patients to an unsupervised take-home regimen that minimized and, in some cases, completely eliminated drug testing requirements [29].

To understand patient and staff views regarding changes in the provision of MOUD during COVID-19 and, in particular, the risks/benefits derived from the decision to increase take-home doses while significantly reducing or eliminating drug testing, this study recruited 25 active PWID users and 25 MOUD staff drawn from the same two clinics in San Juan. PWID were included if they were 19 years or older, had injected at least once in the past 30 days and were receiving treatment at a MOUD clinic during the COVID-19 pandemic. Staff were included if they had been providing services at one of the clinics when the pandemic emerged. The staff sample included a wide range of positions: Front staff in charge of receiving the patient, nurses administering the medication, drug counselors, social workers, and physicians overseeing the medical supervision of the treatment. During the pandemic, the staff had a wide range of years of experience, from months to decades (see Table 2).

A snowball sampling technique was utilized to recruit both samples. This recruitment method relies on participants assisting the researcher to identify and enroll other prospective study subjects [30]. A flyer with a description of the study was distributed in both clinics, and prospective participants were screened for eligibility via a phone-based questionnaire. Using a combined sample size (N = 50) produced data saturation. There were 6 patients and 14 staff recruited from Clinic A (office-based buprenorphine), and 19 patients and 11 staff from clinic B (OTP clinic).

### Data collection

We administered a semi-structured questionnaire to collect data about the experiences of patients and clinic staff seeking/providing care during COVID-19. While the questionnaires probed study participants on the same topics, questions were modified to reflect their different experiences and positions. The first portion of the questionnaire employed closed-ended sociodemographic questions including gender, race/ethnicity, age, education, income, marital status, homelessness, substance and injection use history, and previous courses of and current duration of MOUD treatment. Data obtained from clinic staff also included total years spent in the MOUD treatment field and current position. The qualitative portion of the questionnaire followed the quantitative questions and relied on open-ended questions to collect data about participants’ perceptions of the risks and potential benefits associated with changes in MOUD home delivery during COVID-19 including the potential for medication diversion risk, fears of and perceived frequency of drug overdose episodes and deaths related to take-home procedures, and the effects on treatment attrition caused by the introduction of take-home doses and the reduction or elimination of drug testing controls essential for monitoring drug intake. Interviews were conducted in Spanish by a native speaker and translated into English for analysis. All participants signed an informed consent. Patients received $50 in cash as compensation for their time and efforts. Most of the staff members refused any compensation for their participation. The study received IRB approval from the University of Nebraska-Lincoln.

### Analytic plan

Sociodemographic variables were analyzed to produce descriptive statistics of the population under study. All audiotaped interviews were transcribed and translated from Spanish to English. The transcripts were analyzed with the qualitative analysis software Dedoose. The authors and two research assistants, working simultaneously and collaboratively, undertook the coding. The team used a code book to standardize coding procedures and to solve coding disagreements. Following an inductive procedure, transcribed interviews were grouped into themes, either preestablished themes contained in our interview guide, or “a priori,” and “emergent” themes drawn inductively from the data [31]. The research team iteratively revised and regrouped these codes until they represented a set of higher-level axial codes describing participants’ MOUD treatment experiences as well. A posterior phase in data analysis used the codes produced in the first analytic phase to identify participants’ risk perceptions that better represented the risks related specifically to the changes introduced during COVID-19 in the provision of treatment, providing a textured account of the multiple ways that participants made sense of and navigated overdose risks and other drug-related harms during the pandemic.

## Results

Similarities and differences in patients’ and staff perspectives on the risks associated with unsupervised take-home dosing during COVID-19 and the strategies staff employed to manage overdose risks emerged from the qualitative analysis. We begin with a description of participant sociodemographics, followed by qualitative analysis of patient’s perspectives, followed by provider perspectives and ending with a discussion of similarities and differences in experiences and perspectives and recommendations for harm reduction policies for future pandemics.

### Characteristics of participating patients and staff

Table 1 presents demographic data for patients. The sample had a mean age of 46 years. More than three quarters (84%) were male, and more than half (64%) had been homeless during the past year. Approximately three quarters (72%) were single, and a similar proportion (84%) had achieved a high school diploma or less. Participants were mostly unemployed (52%) with an annual income of $312. Almost half injected one to three times a day and had been in MOUD treatment for seven years.

**Table 1:** Patients’ sociodemographic background

Participants		
Variable	Full sample (N = 25)	
	Mean (%)	Std. dev.
Age (years)	48	9.0
Male	84.0%	
Homeless	64.0%	
Unmarried	72.0%	
High school or less	84.0%	
Unemployed	52.0%	
Monthly income	$ 312.40	4.7
Inject 1–3 times per day	44.0%	
Years in treatment	7	6.1

Table 2 presents demographic data for staff. Staff had a mean age of 46 years with more than half of the sample (63%) female, 7% had been homeless during the past year and approximately half (55%) were married or in a living arrangement as married. Approximately half (55%) had completed post graduate studies and four in five approximately (81%) were employed and working full time with an estimated monthly income of $3,229. On average staff had worked in MOUD clinics for 13 years, 40% provided mental health services.

**Table 2:** Staff’s sociodemographic background

Staff		
Variable	Full sample (N = 25)	
	Mean (%)	Std. dev.
Age (years)	46	9.5
Female	63.0%	
Have a home	96.3%	
Married	55.6%	
Postgrad studies	55.6%	
Working full time	81.5%	
Monthly income	$ 3,229.10	4.0
Years working in the clinic	13	10.3
Mental health Professional	40.7%	

### Qualitative themes

This study presents patients’ and staff perspectives on the benefits and risks associated with unsupervised take home dosing during Covid-19 and the strategies staff employed to manage overdose risk. Our qualitative analysis identified two overarching themes related todrug diversion and overdose risks and the value of drug- testing. The first section presents the patients’ perspectives, followed by the views of the staff.

### Patients’ perception of the risks of drug diversion in response to take-home dosing during COVID-19

One concern during the pandemic was that the implementation of policies that approved take-home doses and the limitation in the capacity to conduct drug tests could increase the probability that patients would divert personal use to selling methadone and buprenorphine to others,. jeopardizing and increasing substance misuse.

The majority of patients interviewed do not indicate concerns about drug diversion. Patients talked about the importance of adhering to their own treatment in order to avoid the painful effects of heroin withdrawal: “… because I’m really on a pretty high dose, you know what I mean, and if I don’t take it, believe me I feel sick.” While none of the patients mentioned that they had sold their medication, many acknowledged they had “friends” who did and why people would be eager to buy it:

“I didn’t do it [sell the take-home dose], but there are many people who do, even when I stopped taking Methadone, I resorted to buying from people who sold it because they [the Methadone program] kind of cut me off, you know, and I started to feel the withdrawal effects too, and then I had to buy a couple of bottles to get off myself at home.”

### “It didn’t hurt me”: patient perspectives on interrupted drug-testing during COVID-19

Pre-COVID MOUD policies required unscheduled drug testing to ensure that patients were compliant with prescribed medications and to detect whether other illicit substances were being used, which could compromise treatment outcomes. During this period, not only were COVID-19 lockdown and/or distancing measures a concern, but the island also was suffering from an increased number of overdose episodes and deaths caused by the presence of fentanyl in the drug supply.

Some patients did not seem particularly worried that less frequent or even a complete lack of drug testing might tempt them to use their drug of choice while still enrolled in MOUD: “I mean I’ve always tried to have my treatment, you know, that I don’t see myself without treatment, you know, that didn’t hurt me.” However, other patients pointed to an increased risk of overdose deaths due to unknown quantities of fentanyl in the drug supply: “Yes, because there have already been deaths due to fentanyl, that [lack of drug testing] does worry me.” In addition, some patients acknowledged that the lack of testing could be an incentive for substance use: “Because they don’t do the tests, that’s why you use substances. If you know they do, tomorrow they will take your urine, you won’t use because you know they will punish you or they will do something, so it wouldn’t be convenient for you.”

Other patients felt “let down” by the disruptions in drug testing, believing that this choice might have hurt them by removing a check on their drug use behavior:

I think it was a mistake. They should have found a way to continue testing, because there were many of us who fell down [i.e., relapsed], let me emphasize, because they were not taking urine or anything. I understand the thing about the disease, but they could have made an adjustment: “I give you the cup, you take it, from here to there, the bathroom is very big.” Many of us left, I am going to tell you, people who had 20 or 20-something bottles of stay at home fell down. (Clinic B).

### Staff concerns about drug diversion due to unsupervised home dosing

Despite the clinics’ inability to conduct routine urine tests because of a lack of staff and other limitations imposed during the lockdown phase of COVID-19, their staffs were aware that diversion was occurring: “Speaking the truth, there is always someone who does it, that’s how it is, I’m not going to cover the sky with my hands because it happens.”

The perception of staff at both clinics was that most participants might have engaged in drug diversion even before COVID-19 during the period where drug testing and take-home doses were available.

Yes, we were very concerned. We know that there are some participants who may be doing it. We have tried to make different strategies to minimize the risk of that happening, but there are always tricks and things, so it is something that worries us. We were concerned and we are very worried that it could happen. We come up with new strategies when we have our suspicions that this could be happening. So it is a concern that we have now and that we had before as well. (Clinic A).

According to staff, the main motivation for engaging in drug diversion seemed to be financial gain. Lack of jobs or income during the pandemic may have played a role in drug diversion: “Even participants who did not sell their medication before started to sell it because they have nothing, they have no job [during the pandemic]. They can make money and they start to sell, and the count was not being administered at that time.” (Clinic B).

Some staff suggested that drug diversion from take-home doses can also be even a “business” opportunity for some patients:

That is not even all of them, not all, but the majority, I understand that a percentage of these participants see it as a business already, to take the prescription and make money on the street. It is a business for them. I have the prescription, and right now a Suboxone on the street is worth 10 and even 12 dollars, we are talking about 10 and 12 dollars for a participant who takes 40 [units] like that, there you have a month, so they play with the system, they play. Not all of them, because many are committed to the process, they come to change, but there are others who do not, who come to seek monetary benefits. (Clinic A).

### Staff efforts to minimize diversion during COVID-19

To minimize the risk of diversion during COVID-19, when drug tests were eliminated or severely restricted for weeks or months, clinics resorted to continuing with certain controls, such as random phone calls or the verification of lot numbers to make sure they correspond to the medication given, which had already been in place before the pandemic and that did not require the use of drug testing:

If the patient sells it, we have control of that because we always call them to count the medicine. In this clinic we count the envelopes. If we have doubts that this patient is selling the medicine, we call them at random, and they have to bring us the empty envelopes of the medicine with the ones that are there, and we verify it by the lot number. That has always been done in this clinic, and it continued to be done during the pandemic. (Clinic A).

Yet despite the clinics’ efforts to monitor substance use patterns and to control drug diversion, staff recognized that the measures implemented might not be entirely adequate but that clinics were constrained by the limitations imposed by COVID-19: “There can always be fear that it can happen [drug diversion] because we were in a moment where we had to make decisions and we could not have contact with patients” (Clinic B).

Staff differed in the extent to which they were concerned about the increased health risk of medication diversion for their patients noting its negative effects on treatment outcomes:

Physically it was noticeable that they were relapsing, but it is very worrying because there we can see that we have to work not only on the delivery of a drug but also on the adherence to the drug. (Clinic A).

Other staff members expressed few concerns, arguing that unsupervised take-home doses made treatment available in the event of a relapse: “If they relapsed, they had the medication available. What they did with the medication is another thing, but it seems to me from the little that I was able to see, when COVID was over, most did not return having significantly relapsed” (Clinic B).

Some among the staff expressed the view that monitoring patients is not the main role of staff, who should instead focus on delivering therapeutic interventions:

We are not here to monitor them. That question is not asked with a diabetic. Are you worried that a diabetic is selling insulin in the street or that someone who is on Viagra because he needs to have sex with his partner is selling it in the street? Nobody asks you that, because if they sell it or don’t sell it, the therapeutic processes are managed, it doesn’t worry me. (Clinic B).

Staff members agreed with the idea that testing interruptions or a lack of testing might increase patients’ desire to use their drugs of choice: “As long as toxicology is not carried out in any treatment program, then a patient with problematic substance use knows that I will not be monitored and I can use substances, so it can be a risk” (Clinic B).

While most staff members felt that the lack of testing might have encouraged substance use among patients at their clinics, not all staff agreed, noting that for a majority of patients the lack of testing had no effect but that for a relative minority of patients, those sent to the program by the judicial system, the lack of testing might have encouraged them to use their drug of choice more often:

This program is not punitive, so if you test positive it’s not like you’re going to get divine justice, it’s not like that. But we have a group of participants who are under legal pressure; in this group, surely not doing the tests might have been a motivation. In other words, most patients were going to carry on normally, as they know that this program is not a punitive program, but that we make a plan with them. It would have made no difference if they had been tested, they would have used the same amount, the person who was going to use it was going to use it the same way. (Clinic A).

Some staff members suggested that testing disruptions were not the only factor influencing participants’ desire to rely on their drugs of choice. In this view, the new stressors brought by COVID-19 on an already vulnerable population might have played a role as well:

In addition to having a problem, they had the pressure of what was happening, and that was a triggering factor for any person. Imagine a mental health patient, a patient who has problematic substance use. There were those thoughts a million times, as one says. So it increased the fact that they could use or not, that there would be lapses, that there would be relapses, that there would be overdoses. (Clinic A).

To minimize the treatment disruptions caused by the alterations to the drug testing regimes during the pandemic, some staff members suggested personalized risk assessments and the use of follow-up phone calls to patients deemed at risk to monitor drug diversion and treatment compliance: Yes, the concern was genuine, but we tried to prevent it through phone calls. We have patients that we already know are patients who do not have good bottle management, a problematic patient, because we always try to give them a more aggressive follow-up by phone to ensure that the treatment continues” (Clinic B).

Despite staff efforts to manage the risks presented by the disruptions to drug testing, the negative effects on participants were hard to avoid:

We found that after we reinstated them [the drug tests] we saw that participants who had been adherent before there was a change due to the pandemic; when we started to do toxicology tests they had recurrences, they had lapses in consumption, so yes, there was an effect because there are some stressors that you do not work on the level of mental health or physical health, which leads to these recurrences and these lapses, and yes, the participants had these effects. (Clinic B).

While most staff recognized that the changes in drug testing implemented as a result of COVID-19 might have increased the risks of treatment attrition, overdose deaths, and other health-related harms for participants, some staff members expressed doubts about the validity of drug tests in the way they are currently conducted in the context of MOUD: “I believe that doping tests no longer work for medical plans because doping tests should be random and sporadic to measure at some point, but patients can also adapt, because if they come every fifteen days or every month to seek treatment, they can make adjustments to avoid testing positive in the doping test. I think doping tests are overrated” (Clinic A).

However, other staff were more likely to harbor a stricter approach to unsupervised take-home doses during COVID-19 and resented the fact that patients who neither earned nor proved that they were deserving of the take homes would still receive them:

To have the benefit of this treatment, the first thing you have to observe is the behavior. Patients are always told that the behavior is the most important thing. I always tell them, “Conduct is like humility. It opens doors for you because the better you modify your habits, that way it will open the doors for you, that is, you will progress, but if you remain in that negative, even if you have what you have, one day you will lose it because you don’t want to. If you are a person who follows the treatment, this is going to open the door for you. You start with a bottle, and when you come to see you have all of them complete 27, which is depending on how you behave. (Clinic B).

## Discussion

The COVID-19 pandemic in Puerto Rico led to an unprecedented shift from clinic drug distribution and drug testing to unsupervised home dosing and disruption in drug testing. Overall, patients were less concerned about drug diversion than staff. The majority of patients reported that the availability of take-home doses and the relaxation of drug testing had no significant effect on their treatment adherence or engagement. Although some may not have wanted to admit drug diversion efforts during the pandemic, many indicated they felt the medications were helping them and feared the consequences of limiting their own doses to diversion Some patients appreciated the convenience of take-home doses during the Island shut down, but resented the relaxation of drug tests, perceiving the tests were a controlling mechanism that supported their treatment adherence.

In contrast, staff expressed greater concern that drug diversion was occurring and that lack of drug testing was increasing the risk of drug overdose. Both clinics were pragmatic, recognizing their response was limited by the constraints imposed by COVID-19 and the need to follow newly issued regulations to provide services during the pandemic. Yet, scale, organizational culture and treatment ideology shaped not only drug diversion risk perception and management strategies but their overall attitude to unsupervised take-home dosing.

While this study on drug diversion risks is novel, there is empirical evidence suggesting that despite a general framework establishing guidelines for unsupervised take-home doses during COVID-19, important differences in the way clinics implemented this measure can be found. For example, a large survey conducted by Livingston et al. among MOUD clinics in the Veteran Hospital Services showed that while methadone and buprenorphine delivery was affected, some patients tended to receive better coverage than others [32]. Another survey conducted with patients receiving take-home deliveries at SAMHSA during the pandemic found wide variability, with some patients receiving their at-home doses while others were still required to dose on site, increasing the risk of COVID-19 transmission [33].

Although the variability described by Livingston and Brothers were found within one large institution, other studies found that important differences in take-home-dose permission, risk perception, and management also exist across clinics. Even within Puerto Rico, a recent study found differences in the ways three community-based clinics responded to the challenges of providing treatment, including unsupervised take-home doses and limited capacity for drug testing [34]. The deficit in drug testing capacity reflects a larger weakness in the available heath infrastructure to support MOUD in Puerto Rico. OTPs are provided by a federally licensed facility through SAMHSA and cover more than 5000 patients [35]. According to recent estimates, office-based buprenorphine reaches 10,000 patients and is dispensed through a combination of prescriptions filled at pharmacies and comprehensive clinics that provide drug counseling, mental health services, and other forms of support while also monitoring patients’ adherence. Most patients are covered through Medicare/Medicare, with only a minority relying on private insurance. Treatment is concentrated in the capital, San Juan, its metropolitan area, and other large cities, with a paucity of medically assisted treatments in rural locations.

While the regulatory framework to access MOUD should be made more flexible in order to expand treatment access during any future pandemic [36, 37], there is the potential for well-intentioned measures like standardized unsupervised take-home doses to unintentionally erect new barriers to treatment, increasing overdose risks and other drug-related harms for an already vulnerable population. This is particularly relevant for Puerto Rican PWID, where many risks for overdose deaths, from economic crisis and despair, to a lack of MOUD [38] syringe exchange providers, to incarceration rates for what are often nonviolent drug-related crimes [39] are magnified. The arrival of fentanyl on the island found a population that had already been significantly affected by a string of natural and economic disasters, including financial collapse during the summer of 2017, when the island was forced to declare bankruptcy and was placed by the US Congress under the administration of a fiscal board charged with managing its economy, and the back-to-back devastation of hurricanes Irma and Maria in the fall of 2017 [40].

While standardization policies provide a framework to provide unsupervised take-home doses during COVID-19, more attention should be paid to the particular local contexts and institutional cultures in order to understand not only their responses to the risks of drug diversion in unsupervised take-home dosing but, more generally, to understand the challenges they faced during COVID-19. Future pandemic preparation for MOUD should take into consideration not only the social contexts in which staff and patients seek and provide care but also, and critically, their lived experiences, perspectives, and knowledge. Failing to consider the views of vulnerable PWID patients when contemplating policy changes constitutes a form of injustice, devaluing the group’s experiences and knowledge and contributing to its stigmatization [41]. Input from the affected population has shown to improve not only the quality of policy recommendations but also, crucially, policy adoption [42, 43] Frontline staff members at MOUD clinics are often rendered invisible in public health policy, which is often created by administrators who are not themselves responsible for the day-to-day implementation of crisis-driven treatment modifications. COVID-19 has required significant changes in MOUD services due to lack of adequate resources, need for quarantining and social distancing, and other institutional decisions affecting the standard of care.

### Limitations

This study is based on a relatively small convenience sample, making the results difficult to generalize to other locations beyond Puerto Rico. Another limitation is that responses might be affected by desirability bias, particularly among patients who were active users. These patients might have underestimated the extent to which drug diversion occurred in the context of unsupervised take-home dosing or underestimated its effects on treatment adherence and engagement. In turn, it is conceivable that staff responses were influenced by desirability bias, tending to overestimate the efficacy of the measures they adopted to manage diversion risks. Nevertheless, we believe that the present study offers novel data on how minority patients and staff at two MOUD clinics perceived the risks associated with unsupervised take-home doses and the strategies they enacted to manage these risks.

## Conclusion

This study underscores the importance of insider perspectives on harm reduction changes in policy implemented during a health crisis. This study shows that while most patients were not concerned about the possibilities of drug diversion during COVID-19, staff at both clinics not only expressed concerns but also attempted to manage risks by profiling and monitoring patients even given the limitations they faced during the lockdown phase of the pandemic. Of note is the finding that staff disagreed among themselves regarding the potential harms of diversion and changes in drug testing, and these different perspectives are important to address so that future pandemic policies are successfully designed and implemented. Our study also illuminates disagreement in risk assessments between patients and providers. This suggests that treatment plans include enhanced communication about these risks within the context of patients lived experience.

## Data Availability

Beyond the excerpts of the transcripts relevant to the study that are available within the paper, full transcripts cannot be shared publicly.

## Abbreviations

HCV: Hepatitis C virus
HIV: Human immunodeficiency virus
MOUD: Medication for OUD
OUD: Opioid use disorder
PWID: People who inject drugs
SAHMSA: Substance abuse and mental health
